# Atomic-Resolution Imaging of Micron-Sized Samples Realized by High Magnetic Field Scanning Tunneling Microscopy

**DOI:** 10.3390/mi14020287

**Published:** 2023-01-22

**Authors:** Weixuan Li, Jihao Wang, Jing Zhang, Wenjie Meng, Caihong Xie, Yubin Hou, Zhigang Xia, Qingyou Lu

**Affiliations:** 1 College of Metrology and Measurement Engineering, China Jiliang University (CJLU), Hangzhou 310018, China; 2 Anhui Province Key Laboratory of Condensed Matter Physics at Extreme Conditions, High Magnetic Field Laboratory, Hefei Institutes of Physical Science, Chinese Academy of Sciences, Hefei 230031, China; 3 High Magnetic Field Laboratory of Anhui Province, Chinese Academy of Sciences, Hefei 230031, China; 4 Anhui Laboratory of Advanced Photon Science and Technology, University of Science and Technology of China, Hefei 230026, China; 5 Hefei Science Center, Chinese Academy of Sciences, Hefei 230031, China

**Keywords:** Scanning tunneling microscopy, high magnetic field, micron-sized sample

## Abstract

Scanning tunneling microscopy (STM) can image material surfaces with atomic resolution, making it a useful tool in the areas of physics and materials. Many materials are synthesized at micron size, especially few-layer materials. Limited by their complex structure, very few STMs are capable of directly positioning and imaging a micron-sized sample with atomic resolution. Traditional STMs are designed to study the material behavior induced by temperature variation, while the physical properties induced by magnetic fields are rarely studied. In this paper, we present the design and construction of an atomic-resolution STM that can operate in a 9 T high magnetic field. More importantly, the homebuilt STM is capable of imaging micron-sized samples. The performance of the STM is demonstrated by high-quality atomic images obtained on a graphite surface, with low drift rates in the X–Y plane and Z direction. The atomic-resolution image obtained on a 32-μm graphite flake illustrates the new STM’s ability of positioning and imaging micron-sized samples. Finally, we present atomic resolution images at a magnetic field range from 0 T to 9 T. The above advantages make our STM a promising tool for investigating the quantum hall effect of micron-sized layered materials.

## 1. Introduction

Scanning tunneling microscopy (STM) has been a powerful tool in studying the atomic structure on material surfaces since its invention [1]. Traditional STMs are capable of working at room temperature [2,3], in liquid [4,5], at low temperature [6,7], and in ultra-high vacuum conditions [8,9] in order to investigate different physical and chemical properties. With the rapid development of material science, small-sized samples in micron dimension are widely synthesized and studied [10,11,12]. As is known, graphene has attracted a significant amount of interest from researchers [13,14]. In recent years, the discovery of magic-angle bilayer graphene [15,16,17] has further improved the research enthusiasm for few-layer materials. The large-sized single crystal sample is quite suitable for atomic-resolution imaging by an STM. While most of the few-layer materials are manufactured in micron size, some are manufactured at the nanometer scale; thus, the STM must be equipped with an X–Y motor to search and image the sample. Many important physical phenomena, such as quantum hall effect [18,19], are found in few-layer materials under high magnetic field conditions. Although a certain amount of STMs working at high magnetic fields [20,21,22] have been reported, none of them have demonstrated the ability of positioning and imaging micron-sized sample. Very few reported STMs [23,24] are equipped with an X–Y motor and have the ability to image small-sized samples. Meanwhile, most X–Y motors [25] are designed at a very large scale, which increases the final outer diameter of the STM and means they cannot be transplanted into a magnet with a small-sized aperture. Thus, constructing an atomic-resolution STM capable of working at a high magnetic field and of positioning the micron-sized sample is of extreme importance.

Designing and constructing a stable STM with the ability to image micron-sized samples has several challenges. Firstly, the material used for building the STM head should have a low coefficient of thermal expansion; the lower the better. Using materials with a high coefficient of thermal expansion, such as copper or stainless steel, will greatly increase the drift rate of the tip-sample mechanical loop, which further degrades the imaging quality of the STM. Secondly, the field of vision between the tip holder and the sample holder should be as wide as possible, or it will be very difficult to position the tip on the central position of the sample. Thirdly, the size of the scanner should be as small as possible. Compared with a large-sized piezo tube, a small-sized piezo tube provides higher imaging stability. Furthermore, to prevent the interference generated from the motor entering the tip-sample junction, the scanner should be separated from the motor during the imaging process. In general, the motor is driven by high-voltage signals, which will inevitably introduce high-voltage signal interference. Full low-voltage scanning signals are more helpful in obtaining high-quality atomic images. Finally, to work at high magnetic field conditions, all materials used in the STM head should be nonmagnetic.

In this work, we present the detailed structure of a stable STM that is capable of atomic-resolution imaging of micron-sized samples. Benefiting from the nonmagnetic design, the homebuilt STM is also capable of atomic-resolution imaging at high magnetic field conditions. To increase the imaging stability of the STM, we use tantalum and sapphire as the main materials to build the STM head. An inertial piezoelectric motor [26] is used to push the sample forward. When detecting the tunneling current, the motor retracts and physically separates from the scanner. The excellent performance of our STM is demonstrated by its low drift rates in the X–Y plane and Z direction, as well as the high-quality atomic images obtained on the graphite surface. To prove the STM’s ability of positioning and imaging micron-sized samples, a 32-μm graphite flake was imaged with atomic resolution. The images were obtained at magnetic fields ranging from 0 T to 9 T, showing the STM’s good immunity to high magnetic fields. The above results make our STM a promising device to investigate the novel properties of micron-sized sample.

## 2. Materials and Positioning Method

The tip used for imaging large-sized graphite is made from a 0.25-mm-diameter Pt/Ir wire (Pt: Ir = 90:10, annealed, from Goodfellow Cambridge Limited) using scissors; the angle between the Pt/Ir tip and the scissors varied from 35° to 45°. One property of Pt/Ir wire is its relative stability; it does not easily oxidize in air, thus a Pt/Ir tip can be used for several months. However, the radius of the hand-cut Pt/Ir tip is very large, making it unsuitable for positioning a micron-sized sample. Compared with the hand-cut Pt/Ir tip, the radius of an electrochemically etched tungsten tip can be as small as several nanometers. With the sharp tungsten tip, we can easily position the tip at the center of a micron-sized sample. To fabricate the sharp tungsten tip, we used an alternating current power supply to perform an electrochemical reaction. One electrode of the power supply was connected to a 0.3-mm-diameter Pt wire, which was immersed in the electrochemical reaction solution. Another electrode and tungsten tip were both connected to the ground. The proper working voltage ranged from 10 V to 20 V. The purity of the tungsten wire was as high as 99.995%. The concentration of NaOH solution used for the electrochemical reaction was 1.0 mol/L. 

To position the sharp tungsten tip on a micron-sized sample, the tip holder and sample holder of our STM head were specially designed. The tip holder was located at one end of the tantalum frame and machined into a U-shaped structure to increase the view. The micron-sized graphite flake was transferred onto the surface of a conductive carbon double-sided adhesive (SPI#05081-AB, from USA), which was then attached to a moveable sapphire piece. The moveable sapphire piece was fixed on the sample holder via conductive paint (SPI#05002-AB, from the USA). The bias voltage was applied on the conductive carbon adhesive via a 0.04-mm-diameter Pt wire. The tip was grounded using silver conductive paint.

What calls for special attention is that the positioning process should be finished when the distance between the tip and the sample is very small. We first performed the coarse approach to adjust the distance between the tip and the sample, and when the distance was reduced to several microns, the motor stopped. The position of moveable sample piece was then adjusted in the X–Y plane until the tip was on the center of the sample. The silver conductive paint became stable after 3 h. After the preparations were completed, the atomic-resolution imaging process was performed.

## 3. System Design

### 3.1. STM Head

**Figure 1:**
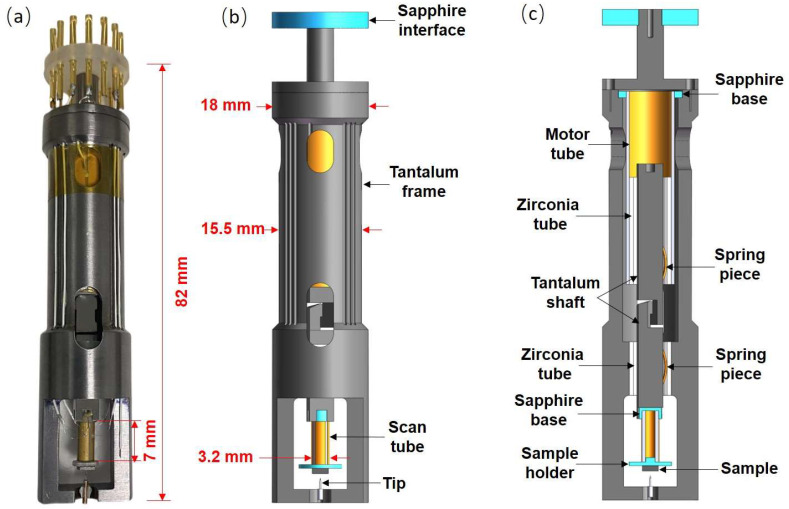
(**a**) Photograph of the STM head. (**b**) Three-dimensional schematic diagram of the STM. (**c**) Section view of the STM head. Detailed parts are displayed in (**b**,**c**).

The STM head mainly consists of the following parts: tantalum frame, inertial piezo motor, scanner, tip holder, sample holder, tantalum shaft, zirconia tube, spring piece, and sapphire base. Figure 1a is a photograph of the STM head, and details of its parts are clearly seen in Figure 1b,c. The motor is constructed using a piezo tube (wall thickness 0.55 mm, EBL#3 type, from EBL Products Inc.) with an outer diameter of 9.1 mm. A zirconia tube is fixed at the free end of the motor tube, and it acts as a guide rail for the tantalum shaft. The tantalum shaft is clamped inside the zirconia tube via a spring piece, which can be moved up and down by applying a pulse signal on the motor tube. The motor tube is fixed at the end of the tantalum frame. The scanner is constructed by using a tiny piezo tube (wall thickness is 0.5 mm, EBL#3 type, from EBL Products Inc.) with an outer diameter of 3.2 mm. The tiny tube is fixed at one end of the lower tantalum shaft via a sapphire base, while the other end of the lower tantalum shaft is connected to the upper tantalum shaft via a pair of hooks. The sample holder is fixed at the free end of the tiny tube via silver conductive paint (SPI#05002-AB, from the USA). The tip holder is located at the opposite position of the sample holder. The sapphire interface is used to fix the signal wires that connect with the piezo tube and tip, and the sample is grounded. When the distance between the tip and the sample is large, the motor is started in order to perform the coarse approach. The upper tantalum shaft, actuated by the motor piezo tube, will push the lower tantalum shaft downwards; thus the scanner moves closer to the tip. Detailed descriptions regarding the motor can be seen in our previous study [27]. When the distance between the tip and the sample is smaller than 1 nm, the tunneling current will be detected by our pre-amplifier, enabling the atomic-resolution imaging process to begin.

### 3.2. High Magnetic Field System

The high magnetic field condition is provided by a cryogen-free superconducting magnet manufactured by Oxford Instruments. The magnetic field strength of the device ranges from 0 T to 12 T. As is known, the vibration generated by the cold head of the magnet is rather large, severely affecting the atomic imaging. To prevent the huge vibration from entering into the tip-sample junction, our STM head hangs at the bottom of a rod with spring damping. The inner diameter of the magnet aperture is as small as 50 mm, which is large enough that the STM head does not contact the inner wall of the magnet. Although the maximum magnetic field is 12 T, a maximum magnetic field lower than 10 T is commonly used to protect the magnet. Furthermore, the magnet provides a room temperature condition of 300 K. Compared with low temperature, atomic-resolution imaging at room temperature remains a greater challenge, and makes it more suitable to prove the stability of the STM.

### 3.3. STM Controller and Pre-Amplifier 

The STM controller was constructed by ourselves. The data acquisition card was bought from National Instruments (model PXI-7852R) and has a high sampling rate of 1 MHz. The PXI-7852R card provides eight low-voltage output ports (−10 V to + 10 V) and eight low-voltage input ports (−10 V to + 10 V). With the help of our homebuilt high-voltage circuits, the maximum output voltage is as high as 200 V, which is high enough to drive the inertial motor. The base noise of the low-voltage and high-voltage output ports are as low as 5 mV and 10 mV, which is mainly achieved by using the four-step filtering of the A/D ports of the data acquisition card, as well as setting the low bandwidth of the filter. Furthermore, all analog circuits were supplied with linear regulated power, which helps reduce noise levels. The software that controls the signal output and data acquisition was written using the LabVIEW platform, enabling different waveforms to be used to control different piezo motors and scanners. The pre-amplifier was constructed on a sapphire board to decrease the leakage current. A 100 MΩ feedback resistance was used to amplify the nA-level tunneling current. To further reduce the outer interference, the bandwidth of our pre-amplifier circuits was limited to as low as 200 Hz.

## 4. Performance Test

**Figure 2:**
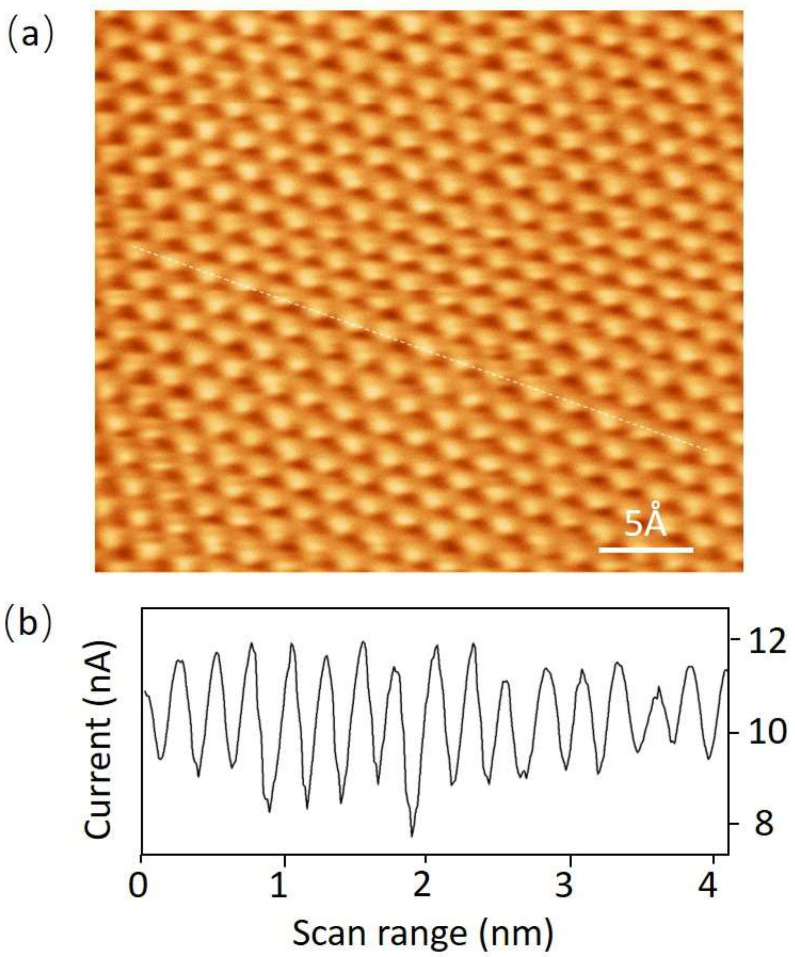
(**a**) Atomic-resolution image of large-sized graphite obtained with constant height mode. The average tunneling current is 10 nA and the scan speed is 7 lines/s. (**b**) The tunneling current profile obtained along the white line in (**a**).

With the new STM, the imaging resolution was first tested at room temperature. Figure 2a is a high-quality STM image with atomic resolution; the data were acquired under constant height mode, with an average tunneling current of 10 nA. Figure 2b shows the tunneling current along the white line in Figure 2a. Because of its advantage of high scan speed, constant height mode was chosen to perform the atomic-resolution imaging. When scanning a large area, the constant current mode is more suitable for its feedback mechanism. A high-quality STM image and tunneling current profile showing the high imaging precision of the new STM are shown in Figure 2.

**Figure 3:**
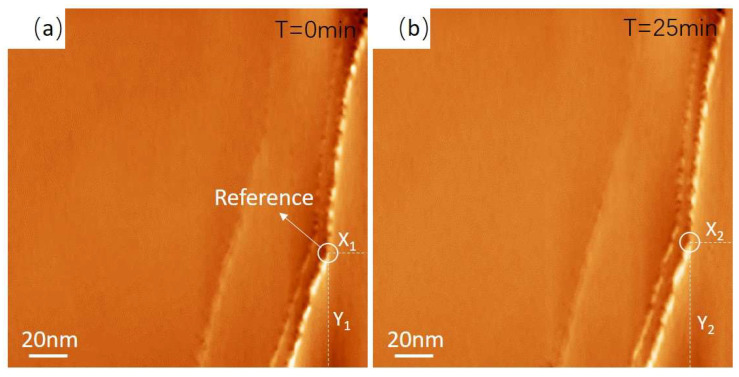
(**a**) Large area topography of large-sized graphite obtained with constant current mode; the reference dot is marked by a white circle. The tunneling current is 2.5 nA, and the scan speed is 1 lines/s. (**b**) Topography obtained by scanning the same area after 25 min.

To quantitatively characterize the imaging stability of the newly built STM, drift rate measurements were taken. The drift rate in the X–Y plane was measured by scanning the same area repeatedly. As seen in Figure 3a,b, the reference dot marked by a white circle moves a very small distance: ΔX = X_1_ − X_2_ = 2.1 nm, ΔY = Y_1_ − Y_2_ = 3.7 nm, thus we can obtain the drift rates of 84 pm/min and 148 pm/min in the X direction and Y direction, respectively. The drift rate in the Z direction was measured by acquiring the distance variation between the tip and the sample; the calculated drift rate in the Z direction is 76 pm/min. The drift rates of reported room temperature STM mainly range from 84 pm/min to 150 pm/min [28,29], which is much larger than our newly built STM.

**Figure 4:**
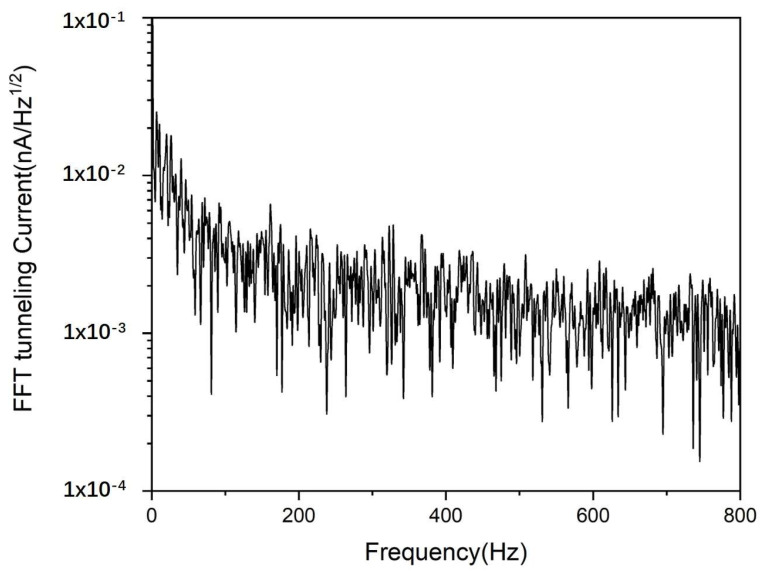
Base noise obtained by acquiring the tunneling current with feedback loop open. The tunneling current is set as 2.5 nA.

Figure 4 shows the base noise of the STM obtained by acquiring the tunneling current with the feedback loop open, and the tunneling current is set at 2.5 nA. The data presented in Figure 4 were processed using the FFT function of Origin software. As seen in the figure, no significant vibration enters the tip-sample junction, which greatly promotes the high-quality STM imaging.

**Figure 5:**
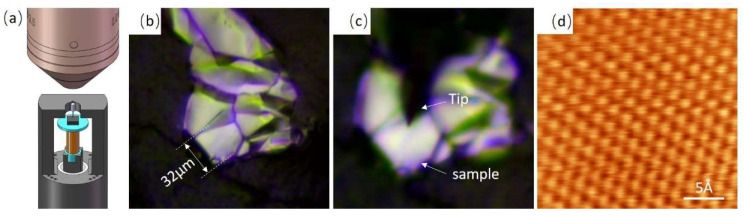
(**a**) Three-dimensional schematic diagram of the positioning process. (**b**) Optical microscope photograph of the small-sized graphite flake before the positioning process. (**c**) Optical microscope photograph obtained after the positioning process. (**d**) Atomic resolution image obtained on micron-sized sample with constant height mode; the average tunneling current is 12.5 nA, and the scan speed is 10 lines/s.

Figure 5a shows a schematic diagram of the positioning mechanism, in which the optical microscope, tip, and sample are clearly seen. Figure 5b is an optical photograph obtained before the positioning process; the size of the target graphite flake is 32 μm. Figure 5c is an optical photograph obtained after the positioning process; the front end of the tip is now at the center position of the graphite flake. When the positioning process is finished, the atomic-resolution imaging of the graphite flake is performed immediately. Figure 5d is the STM image obtained with constant height mode; the average tunneling current is 12.5 nA. The above results prove the high-precision positioning and atomic resolution imaging ability of our STM device.

**Figure 6:**
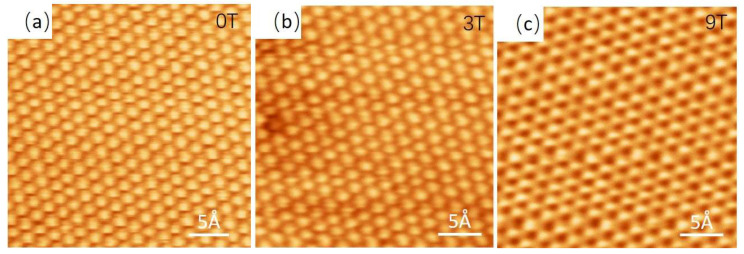
Atomic resolution images of graphite obtained at magnetic field of (**a**) 0 T, (**b**) 3 T, and (**c**) 9 T. The average tunneling current is 12.5 nA, and the scan speed is 10 lines/s.

Finally, the STM device was tested in a cryogen-free superconducting magnet with huge vibration interference. Its excellent immunity to the high magnetic field and huge vibration was proved by the continuous atomic resolution images obtained when sweeping the magnetic field from 0 T to 9 T, as seen in Figure 6a–c. The high-quality images prove that the atomic-resolution imaging can be performed in higher magnetic fields.

## 5. Conclusions

In this work, we present the novel design and excellent performance of our newly built STM. The tip holder is designed in a U shape to provide a large viewing field, which is helpful for positioning micron-sized samples. The excellent performance is demonstrated by high-quality STM images obtained on a graphite surface. The low drift rates in the X–Y plane and Z direction further prove the stability of our STM device. Furthermore, the atomic-resolution image obtained on a 32-μm graphite flake shows the device’s good positioning ability of small-sized samples. Finally, we present continuous STM images of graphite at magnetic fields ranging from 0 T to 9 T. The above results demonstrate the STM’s high-precision positioning and imaging ability, as well as its excellent immunity to high magnetic fields. We expect to use this STM to study the rich physical phenomenon of micron-sized materials in the future.

## Data Availability

The data presented in this study are available upon request from the corresponding author.
